# International Nuclear Medicine Consensus on the Clinical Use of Amyloid Positron Emission Tomography in Alzheimer’s Disease

**DOI:** 10.1007/s43657-022-00068-9

**Published:** 2022-08-26

**Authors:** Mei Tian, Chuantao Zuo, Ali Cahid Civelek, Ignasi Carrio, Yasuyoshi Watanabe, Keon Wook Kang, Koji Murakami, Valentina Garibotto, John O. Prior, Henryk Barthel, Yihui Guan, Jiaying Lu, Rui Zhou, Chentao Jin, Shuang Wu, Xiaohui Zhang, Yan Zhong, Hong Zhang, Molecular Imaging-Based Precision Medicine Task Group of A3 (China-Japan-Korea) Foresight Program

**Affiliations:** 1 PET Center, Huashan Hospital, Fudan University, Shanghai, 200235 China; 2 Human Phenome Institute, Fudan University, Shanghai, 201203 China; 3 Department of Nuclear Medicine and PET Center, The Second Affiliated Hospital of Zhejiang University School of Medicine, Hangzhou, 310009 China; 4 National Center for Neurological Disorders and National Clinical Research Center for Aging and Medicine, Huashan Hospital, Fudan University, Shanghai, 200040 China; 5 Department of Radiology and Radiological Science, Division of Nuclear Medicine and Molecular Imaging, Johns Hopkins Medicine, Baltimore, 21287 USA; 6 Department of Nuclear Medicine, Hospital Sant Pau, Autonomous University of Barcelona, Barcelona, 08025 Spain; 7 Laboratory for Pathophysiological and Health Science, RIKEN Center for Biosystems Dynamics Research, Kobe, Hyogo 650-0047 Japan; 8 Department of Nuclear Medicine, Seoul National University College of Medicine, Seoul, 03080 Korea; 9 Department of Radiology, Juntendo University Hospital, Tokyo, 113-8431 Japan; 10 Diagnostic Department, University Hospitals of Geneva and NIMTlab, University of Geneva, Geneva, 1205 Switzerland; 11 Department of Nuclear Medicine and Molecular Imaging, Lausanne University Hospital, Lausanne, 1011 Switzerland; 12 Department of Nuclear Medicine, Leipzig University Medical Center, Leipzig, 04103 Germany; 13 Key Laboratory of Medical Molecular Imaging of Zhejiang Province, Hangzhou, 310009 China; 14 The College of Biomedical Engineering and Instrument Science of Zhejiang University, Hangzhou, 310007 China; 15 Key Laboratory for Biomedical Engineering of Ministry of Education, Zhejiang University, Hangzhou, 310007 China

**Keywords:** Positron emission tomography (PET), Alzheimer’s disease, Amyloid, Brain imaging, Expert consensus

## Abstract

Alzheimer’s disease (AD) is the main cause of dementia, with its diagnosis and management remaining challenging. Amyloid positron emission tomography (PET) has become increasingly important in medical practice for patients with AD. To integrate and update previous guidelines in the field, a task group of experts of several disciplines from multiple countries was assembled, and they revised and approved the content related to the application of amyloid PET in the medical settings of cognitively impaired individuals, focusing on clinical scenarios, patient preparation, administered activities, as well as image acquisition, processing, interpretation and reporting. In addition, expert opinions, practices, and protocols of prominent research institutions performing research on amyloid PET of dementia are integrated. With the increasing availability of amyloid PET imaging, a complete and standard pipeline for the entire examination process is essential for clinical practice. This international consensus and practice guideline will help to promote proper clinical use of amyloid PET imaging in patients with AD.

## Introduction

As the leading cause of dementia in people older than 65, diagnosing and managing Alzheimer’s disease (AD) is tricky (Scheltens et al. 2021). However, according to the classic amyloid (Aβ) cascade hypothesis, Aβ-related toxicity is the primary cause of synaptic dysfunction and subsequent neurodegeneration that underlies the progression characteristic of AD (Hardy and Selkoe 2002; Hardy and Higgins 1992). In 2018, the National Institute on Aging and Alzheimer’s Association (NIA-AA) summarized the three significant dimensions of pathologic mechanisms as A (amyloid) / T (phosphorylated tau) / N (neurodegeneration). Furthermore, they ruled out those with negative amyloid from the Alzheimer’s continuum, highlighting various pathological biomarkers’ roles for accurate disease characterization and understanding (Jack et al. 2018).

Molecular imaging is a field of medical imaging which enables visualization, characterization, and measurement of biological processes at the molecular and cellular level in vivo (Weissleder. 1999). The development of molecular imaging has shown great potential to reform traditional pathology, and may lead to a new pattern of pathological practice termed transpathology (Tian et al. 2022, 2021). Positron emission tomography (PET), the most frequently used molecular imaging tool, has brought a new era in AD management. With the development of ligands which could harbor high affinity to Aβ in vivo and capture insoluble Aβ fibrils in plaques accurately, amyloid status could be visualized. In AD patients, the accumulation of Aβ might start about 20 years before the onset of dementia (Gordon et al. 2018; Villemagne et al. 2013). A negative amyloid PET diminishes the likelihood that a patient’s cognitive impairment is due to AD. However, in patients with cognitive impairment and positive amyloid PET findings, in addition to AD, the differential diagnosis includes dementia with Lewy bodies (Arnaoutoglou et al. 2019; Ferreira et al. 2020), cerebral amyloid angiopathy (Brenowitz et al. 2015; Greenberg et al. 2020) and amyloid positivity concomitant to any other brain disorders. Besides, an association was reported between elevated brain amyloid and subsequent cognitive decline (SCD) among cognitively ‘normal’ persons (Donohue et al. 2017). Therefore, the findings of amyloid PET are not sufficient for a definitive diagnosis of AD.

In 2013, the Alzheimer’s Association (AA) and the Society of Nuclear Medicine and Molecular Imaging (SNMMI) released the Appropriate Use Criteria (AUC) for amyloid PET imaging suggestions and recommendations (Johnson et al. 2013a, b). This international consensus report aims to analyze and update the fundamental aspects of amyloid PET in a clinical setting, including the clinical indications and contraindications, radiosynthesis of the radiopharmaceuticals, patient preparation, acquisition and interpretation of PET images.

In January 2021, The Molecular Imaging-based Precision Medicine Task Group of A3 (China-Japan-Korea) Foresight Program convened an international panel of researchers and clinicians with expertise in amyloid PET molecular imaging and Alzheimer’s Disease, comprising members from across China, US, Spain, Japan, Korea, Switzerland, Germany, to discuss the issues relating to the application of amyloid PET in the medical settings of cognitively impaired individuals. The panel was then divided into subgroups to review literature and generate content of various sections in the manuscript, focusing relevant published guidelines and clinical experience on amyloid PET from 1 January 2013 to 1 February 2022. Articles not identified in screening but deemed relevant by authors were also included. A draft manuscript was written and circulated among authors to collect additional information, comments and recommendations, by which the manuscript was iteratively revised. The final manuscript represents broad agreement on principles to which all authors could subscribe.

## Clinical Indications

The 2013 AUC proposed three clinically appropriate indications and seven inappropriate indications for the clinical application of amyloid PET (Johnson et al. 2013a) (Table 1). These criteria are justified by a large prospective multicenter trial (Imaging Dementia–Evidence for Amyloid Scanning, IDEAS) (Rabinovici et al. 2019). By selecting individuals based on the 2013 AUC, the amyloid PET findings brought a change in disease management in a majority of engaged individuals. In addition to the 2013 AUC, we suggested considering the following clinical scenarios.

**Table 1:** Recommend indications of amyloid PET

Amyloid PET imaging is appropriate for
(1) Patients with mild cognitive impairment, in whom clinical uncertainty exists
(2) Patients with a dementia syndrome suggestive of AD, but with an atypical presentation or suspected mixed cause
(3) Patients with early-onset progressive cognitive decline (usually defined as 65 years or less in age)
(4) Patients meeting clinical core criteria for a probable diagnosis of AD when the information on amyloid status is required for management^a^
(5) Patients with a diagnosis of SCD meeting the core clinical criteria for SCD-plus^a^
Amyloid PET imaging is inappropriate for
(6) Determining dementia severity
(7) The study solely based on a positive family history of dementia or presence of APOE ε4
(8) Patients with a cognitive complaint that is unconfirmed by clinical examination
(9) Instead of genotyping for suspected autosomal mutation carriers
(10) Asymptomatic individuals
(11) Nonmedical usage (e.g., legal, insurance coverage, or employment screening)

*PET* Positron emission tomography, *AD* Alzheimer’s disease, *SCD* Subjective cognitive decline, *APOE* Apolipoprotein E

^a^Updated indications on appropriate use criteria (AUC) for Amyloid PET imaging posited by the Alzheimer's Association and the Society of Nuclear Medicine and Molecular Imaging launched in 2013 (Donohue et al. 2017; Johnson et al. 2013a)

### Patients with a Clinical Diagnosis of Probable AD

In two phase III trials of bapineuzumab, some patients who met the criteria (McKhann et al. 1984) of probable AD did not reach the positive threshold for amyloid-positive category (6.5% of carriers of the apolipoprotein E (APOE) ε4 allele, 36.1% of noncarriers) (Salloway et al. 2014), suggesting that the clinical diagnosis is unreliable to identify underlying pathology, especially when the care-providing physicians are general practitioners rather than dementia specialists.

There are wide variations in the accuracy of the clinical diagnosis of AD, nationally and internationally (Johnson et al. 2013a), and the previous Candian Consenesus guidelines suggest that the ordering of an amyloid PET should be limited to dementia specialists (Laforce et al. 2016). Given the ongoing development of amyloid-targeting therapy recently, it is necessary to identify the amyloid status in patients with clinical diagnosed probable AD before giving such prescription if available. Therefore, we recommend modifying the original inappropriate indications # 4 of the 2013 AUC: “Patients with core clinical criteria for probable AD with a typical age of onset” to be an additional appropriate indication (4) patients meeting clinical core criteria for a probable diagnosis of AD when the information on amyloid status is required for patient’s management.

### Patients with a Diagnosis of Subjective Cognitive Decline (SCD) Meeting the Core Clinical Criteria for SCD-Plus

SCD has been proposed to be the first possible symptomatic expression of preclinical AD (Jessen et al. 2014; Sperling et al. 2011). Although patients with SCD may experience a subjective cognitive decline, there is usually no documentable objective cognitive impairment on clinical assessment. A community-based and memory-clinic settings multicenter study found that although most subjects with SCD did not develop any dementia and remained cognitively normal, some patients progressed to have dementia during the following-up period. Among those with SCD who went to dementia, two-thirds were attributable to AD dementia, and the remaining developed other subtypes of dementia (Slot et al. 2019). Consequently, an “SCD-plus criteria” has been proposed as an improvement strategy for the likelihood of preclinical AD in individuals with SCD (Jessen et al. 2014).

According to the proposed framework by SCD-I Working Group (Jessen et al. 2014), the SCD-plus criteria are (1) subjective decline in memory, rather than other domains of cognition; (2) onset of SCD within the last five years; (3) age at onset of SCD ≥ 60 years; (4) clinically suspected SCD; (5) feeling of worse performance than peers (here operationalized with the specific questions in the Cognitive Change Index (CCI) questionnaire); (6) confirmation of perceived cognitive decline by a close relative or friend; and (7) APOE ε4 alleles carriership. In addition to these preliminarily defined features, these criteria also mention the importance of obtaining biomarker evidence for AD (defined as preclinical AD) if possible. Though still limited, a Subjective Cognitive Impairment Cohort (SCIENCe) study has found that the SCD-plus criteria age ≥ 60 and APOE ε4 carriership were associated with an increased risk of preclinical AD, which is defined by amyloid positivity on either PET or cerebrospinal fluid (CSF) (Slot et al. 2018). Another study observed that the following SCD-plus features were associated with lower Aß-42 levels in CSF: Onset of subjective decline within five years; Confirmation of cognitive decline by a close relative or friend, and decline-related worries (Miebach et al. 2019). These two independent studies validated the current SCD-plus features as predictors of AD pathology. Further, they laid the foundation for advancing the disease intervention of the preclinical AD stage to those who meet SCD-plus criteria with the positive amyloid result. While the date for amyloid positivity percentage in individuals with SCD-plus is unavailable, a recent study based on the data from the multicenter memory clinic-based DZNE (German Center for Neurodegenerative Diseases) Longitudinal Cognitive Impairment and Dementia project reported a close to 40% amyloid positivity in general SCD persons from the clinical settings (Jessen et al. 2022). Another multicenter study of 1640 persons with SCD found that beyond the large variability in the frequency of amyloid positivity between cohorts (10–76%), age, settings, APOE-ε4 carriership and SCD-specific characteristics may facilitate the identification of amyloid-positive individuals, which is consistent with the definition of SCD-plus (Janssen et al. 2021). Therefore, it’s reasonable to suspect the number in individuals with SCD-plus would be higher than these reported ones. To this end, we recommend listing one more indication of amyloid PET: patients with core clinical measures for SCD-plus. The summary of updated clinical presentations is listed in Table 1.

## Radiopharmaceuticals

Carbon 11 (^11^C)–labeled Pittsburgh compound B (PiB) is the initially developed PET radiotracer for amyloid imaging, which crosses the blood–brain barrier, binds to amyloid with high affinity, and rapidly clear from normal-gray matter (Klunk et al. 2004). However, the clinical and research utilization of ^11^C-PiB PET is limited mainly by the 20 min short half-life of ^11^C. Subsequently, several amyloid ligands, labeled by fluorine 18 (^18^F) with a longer half-life (110 min), have been developed, allowing the more extensive practice. To date, three ^18^F-labeled amyloid PET ligands are available: florbetapir (^18^F-AV-45, Amyvid), flutemetamol (^18^F-GE067, Vizamyl), and florbetaben (^18^F-BAY94-9172, NeuraCeq), and all of them are approved by the U.S. and the European authorities. Besides, another ^18^F-labeled amyloid PET ligand—NAV4694 (^18^F-AZD4694)—is also used in the clinical trial and research.

Although these amyloid radiotracers share a common imaging target with similar imaging characteristics, each has different tracer kinetics, specific binding ratios to amyloid, with its unique ideal imaging parameters. Thus, the recommended standards for applying these amyloid compounds are different (Cselényi et al. 2012; Healthcare et al. 2017; McNamee et al. 2009; Rowe et al. 2013; Rowe and Villemagne 2013; Vandenberghe et al. 2010; Villemagne et al. 2011; Wong et al. 2010). The detailed protocols are presented in the next section and Table 2.

**Table 2:** Recommendations for performing amyloid PET with available radiopharmaceuticals

Compound	Recommended dosage (MBq)	Waiting period (min)	Scan period (min)	Recommended Display Color Scale
^11^C-PIB	500	50	20	N.A
Florbetapir (^18^F-AV-45, Amyvid)^a^	370	30–50	10	Grayscale (or inverse grayscale)
Flutemetamol (^18^F-GE067, Vizamyl)^a^	185	60–120	10–20	Rainbow color scale
Florbetaben (^18^F-BAY94-9172, NeuraCeq)^a^	300	45–130	20	Grayscale (or inverse grayscale)
NAV4694 (^18^F-AZD-4694)	200	40	30	N.A

*N.A.* no officially recommendation was available

^a^These three radiopharmaceuticals have been approved by U.S. and the European authorities

## Patient Preparation and Image Acquisition

**Fig. 1:**
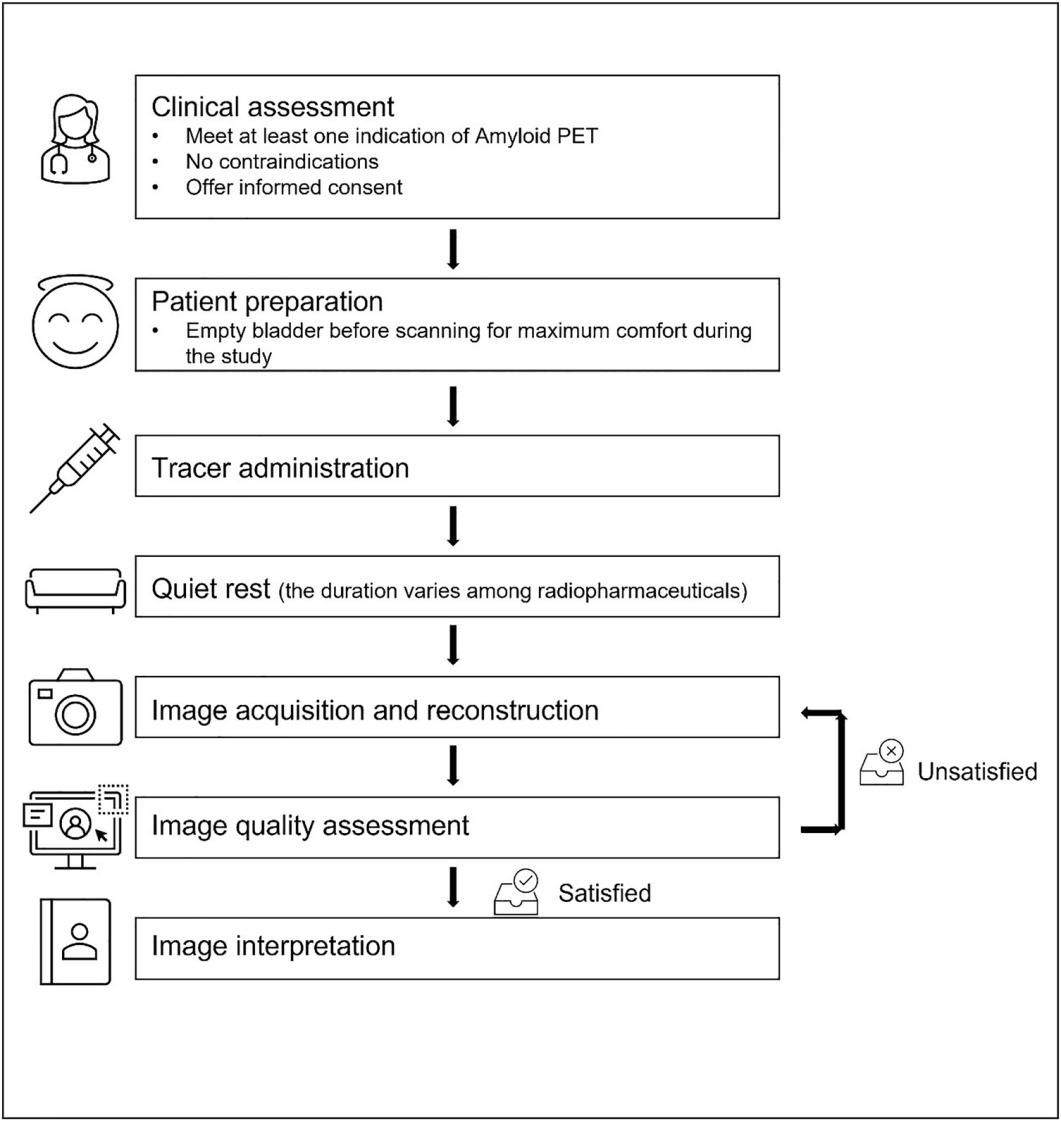
The flowchart of the recommended examination procedures in performing amyloid PET scanning and reporting

SNMMI Procedure Standard and European Association of Nuclear Medicine (EANM) Practice Guideline for amyloid PET Imaging of the Brain 1.0 have offered the reference for amyloid PET scans (Minoshima et al. 2016). In the following paragraphs, we summarized the guidelines for the three FDA approved radiotracers. The Workflow diagram is shown in Fig. 1.

### Patient Preparation

The examination should only be conducted in those who meet one indication of amyloid PET and do not have any contraindications after obtaining the detailed medical history, providing the detailed explanation of the procedure, and obtaining informed consent from the study subjects or their legal guardians. The study subject should empty their bladder for maximum comfort before scanning, though the water and food do not influence amyloid PET. The use of amyloid-targeting drugs before the amyloid PET scan should, of course, be taken into account for the interpretation of the results.

### Tracer Administration

To ensure patient safety and image quality, a visual inspection of the radiopharmaceutical dose is necessary before its administration. The recommended doses are listed in Table 2. The radiotracer should be injected as a single intravenous slow bolus, after which the catheter needs to be flushed.

### Patient Positioning

A supine position with proper head support to reduce the potential head movement is preferred. The patient's extreme neck extension or flexion should be avoided. The entire brain, including the cerebellum as a whole, should be in the field of view.

### Image Acquisition

Following the required uptake phase, amyloid PET images were obtained in 3-D mode, applying appropriate attenuation corrections. Recommended uptake times are presented in Table 2. Image reconstruction is necessary and ordered-subset expectation maximization is the most common reconstruction method in the literature.

### The Safety of Subject

To minimize the risks and protect the human subjects, besides what have been described above, additional measures should be done including: (i) While waiting for the examination after injection, the medical personnel should pay attention to observe whether the examinee is uncomfortable and provide timely help if necessary. (ii) For those with mobility or cognitive impairment who are difficult to cooperate with the examination, dedicated medical personnel should be arranged to provide assistance throughout the examination, and for those unable to cooperate, it should be suggested to suspend the examination. (iii) After the image acquisition, the examinee should be asked to rest quietly in the rest room for a period of time (such as half an hour) to ensure that there is no discomfort before leaving and should be asked to contact the hospital if any discomfort occurs after leaving.

## Qualifications and Responsibilities of Personnel

Both involved physicians and technologists should receive basic training before participating in the amyloid PET imaging process. The SNMMI Procedure Standard & EANM Practice Guideline list the detailed requirements (Minoshima et al. 2016).

## Image Interpretation

The image quality should be assessed as early as possible. If it is significantly degraded by head motion, rescanning or rescheduling the patient should be considered. The image interpretation could be divided into visual assessment and quantitative analysis.

### Visual Assessment of the Amyloid PET Images by Properly Trained Nuclear Physicians and Dementia Experts

General instructions for the image interpretation of the three approved amyloid PET compounds are proposed based on the basic guidelines outlined by SNMMI (Minoshima et al. 2016).

#### Image Display

Typically, a grayscale or inverse grayscale display with 256 or greater shades is preferred. However, for ^18^F-flutemetamol, the manufacturer recommends using a specific color scale, ‘Rainbow color scale’. The transaxial plane is the recommended orientation to display the PET images. The coronal and sagittal planes may contribute to better define the uptake area and ensure the review of the entire brain. The optimum intensity to display images differs among the three compounds. The maximum intensity of the display scale of ^18^F-florbetapir PET images should be the brightest region of the entire brain. However, for ^18^F-florbetaben, images should be adjusted to the white matter maximum, while for ^18^F-flutemetamol, the optimum display intensity should be set to 90% of the pons. The transaxial plane brain images should be displayed from caudal to cephalad, which would help the reviewer identify the normal cerebellar gray-white matter (GM-WM) differentiation; the cerebellum usually is free of amyloid deposits.

Although rare, cerebellar amyloid deposits in AD may occur as in autosomal dominant cases (Cole et al. 1993; Giau et al. 2018; Lu et al. 2021; Shimada et al. 2020) or advanced stage AD (Thal et al. 2002), a careful assessment of the entire cerebral cortex GM for the presence of radiotracer uptake is performed. The reviewer should also evaluate the striatum activity since increased binding is frequently seen on positive ^18^F-flutemetamol scans (Beach et al. 2016), as well as ^18^F-florbetapir and ^11^C-PiB scans in advanced AD patients (Hanseeuw et al. 2018). The interpreting physician should also be aware that owing to its anatomy, cerebellar GM radiotracer intensity is lower than that in the cerebral cortex.

#### Definition of a Negative and Positive Amyloid PET Scan

A study with minimal or no radiotracer binding of the GM and only with nonspecific WM tracer uptake constitutes a negative amyloid PET study. On the other hand, a positive amyloid PET scan shows the loss of clear cerebral GM-WM contrast due to increased tracer uptake in the cerebral GM. The edges of cerebral GM are smooth and regular. Of the three amyloid radiotracers, for ^18^F-florbetapir, a positive scan requires the presence of at least two abnormal (positive) cerebral regions with increased GM uptake blending into WM. For the other two radiotracers, only one positive cerebral cortical region would be sufficient.

The most common disease-involved regions are (i) the lateral temporal and frontal lobes, (ii) the posterior cingulate cortex/precuneus, and (iii) the parietal lobes. In the meantime, the sensorimotor and visual cortex are usually spared from the disease. In subcortical structures, increased tracer uptake is often found in the striatum.

#### Signs of Specific Regions

According to the description of Lundeen et al. (2018), several signs in specific regions can help to distinguish the negativity and positivity of amyloid PET.

(i) Temporal-occipital “ridge” and “plain” signs: The negative study presents a “ridge” in the temporal-occipital region, and the positive one creates a smooth “plain” on the axial images.
(ii) Occipital “kissing hemispheres” sign: Since the medial aspects of the occipital GM harbor no radiotracer binding in the negative study, while such binding exists in the positive ones, a “kissing hemispheres” sign is taken shape on the axial images.
(iii) Frontal “diamond” and “kissing hemispheres” signs: similar to occipital “kissing hemispheres” sign, lacking radiotracer binding to the GM lining the interhemispheric fissure in the negative examination creates a “diamond” sign on the axial images, and the filling of binding in this region creates a “kissing hemispheres” sign in the positive examination. Besides, a “cartoon hand” sign on the axial images is also observed in the negative images formed by white matter tracts. In contrast, such ‘sign’ disappears due to the binding to the GM in the positive images.
(iv) Frontal “tree” sign: When viewing coronally, the filling signals between WM creates a “summer branches of tree sign” in the positive study, while the negative one appears like a “tree in the winter”.
(v) Parietal “double convex lens” and “kissing hemispheres” signs: The negative examination shows a “double convex lens” sign on the axial projection. In contrast, such a ‘sign’ disappeared and was replaced by a “kissing hemispheres” sign, similar to the previously described “kissing hemispheres” sign in occipital and frontal lobes. Such a sign also exists in the precuneus, which involves and continually accumulates amyloid regions in AD. It is vital to observe the precuneus in all three planes due to its thin height and not easily observed location.
(vi) “Striatal gap” and “striatal bridge” signs: As previously mentioned, striatal binding is frequently seen in ^18^F-flutemetamol positive scans, which build a “striatal bridge” in all three planes. On the contrary, the lack of signal in the negative study presents a “striatal gap” sign.
(vii) “Asymmetric pattern”: While traditional theory believes the amyloid deposition usually distributes symmetrically, recently finding posits the asymmetric amyloid pattern is not uncommon in AD spectrum, especially in mild cognitive impairment (MCI) subjects. Furthermore, though the evidence is limited at present, the disappearance of such an asymmetric pattern suggests disease progression (Yoon et al. 2021). Conversely, the negative image should be almost symmetrical, indicating the occurrence of an asymmetric pattern is a vital clue for amyloid positivity.

#### Quality Control

The patient’s unexpected head motion during image acquisition will cause image artifacts. Therefore, if the attempt to reorient into standard alignment using postprocessing software does not work, one would consider rescanning the patient or rescheduling.

Atrophy is another factor that influences the visual assessment of amyloid PET. As an inevitable change during aging, atrophy might generate false-positive and false-negative reports. The former is because of the over-estimation of binding signals in the surviving cortex caused by the spillover effect of neighboring WM, and the latter often happens in those with severe atrophy, where the positive signal in GM is unable to be distinguished from the signal in the adjoining WM due to the smaller volume. In such uncertain cases, coregistered anatomical images (such as computed tomography (CT) and magnetic resonance imaging (MRI)) might assist in the definition of the relative accurate localization of GM-WM uptake. Besides, when an ^18^F-FDG PET or perfusion single photon emission computed tomography (SPECT) scan is available, observed decreased functional regions may help.

#### Data Integration

**Fig. 2:**
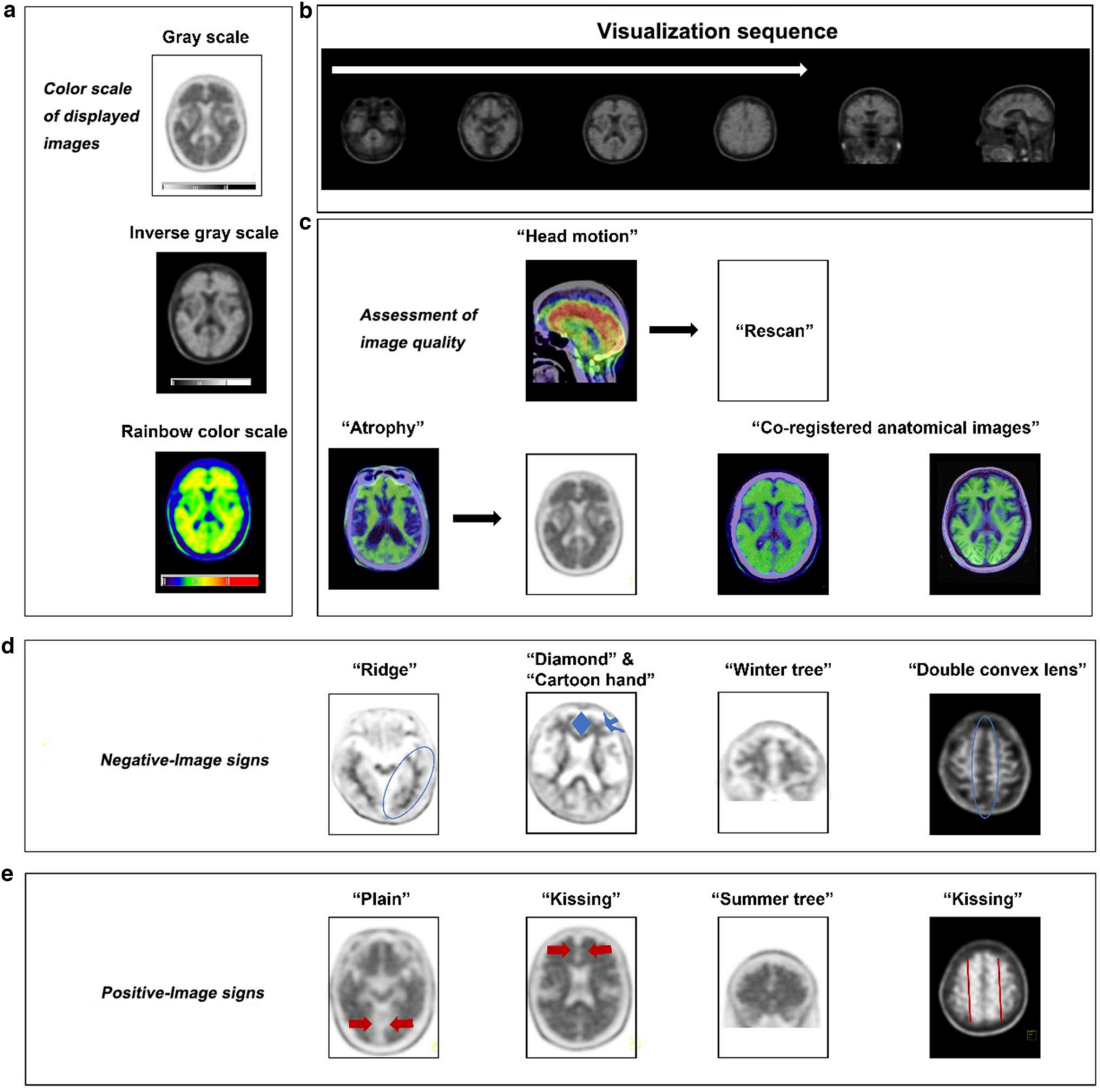
The visual assessment algorithm of amyloid PET images includes color scale of displayed images (**a**), observation sequence (**b**), assessment of image quality (**c**), negative-image signs (**d**) and positive-image signs (**e**)

The final report should integrate all the detected information of the patient, not only by the amyloid PET but also by the other imaging modalities and clinical data. In addition, the interpreting physician should keep the other possible amyloid-positive diseases and typical aging-related positivity in the differential diagnosis. The visual assessment algorithm is summarized in Fig. 2.

### Quantification of the Amyloid PET Images

While visual assessment represents the primary method for image interpretation in clinical setting, various quantification methods have increasingly been used as important adjuncts to visual reading, especially when the degree of amyloid deposition is close to the positive threshold, or images are interpreted by readers lack experience (Bucci et al. 2021). Quantification methods also provide important information to support decision making when the amyloid burden become clinically relavent for disease management. To date, several types of analysis have been used in quantifying amyloid PET, including volume of interest (VOI)-based, voxel-based, and artificial intelligence (AI)-based methods. Among them, the VOI-based quantification, which calculates the mean amyloid retention values in the target VOIs and normalizes the values to a reference region free of amyloid, is the most widely used approach, yielding a variety of semiquantative measures, such as standardized uptake value ratio (SUVR), non-displaceable binding potential (BPND), and distribution volume ratio (DVR). While the calculations of BPND and DVR require dynamic PET scans, SUVR is suitable for static images, making the use of SUVR more prevalent in practice, especially in clinical settings. The voxel-based analysis is similar to the VOI-based method, though the quantification is performed on voxel-wise level instead of VOI level. The AI-based method is applied for the definition of VOIs, cut-off value of positivity and classification, etc.

Before any semi-quantification, it is strongly recommended to spatially normalize the raw brain PET images into the standardized anatomic space like Montreal Neurological Institute (MNI) standard space. And the spatial normalization is recommended to perform based on individual T1-weighted MRI which is acquired during the same period. Take the Statistical Parametric Mapping (SPM, version12, http://www.fil.ion.ucl.ac.uk/spm/software/spm12/) for example, the individual MRI scan should be reoriented to anterior commissure and coregistered to the MNI-152 template. The individual PET scan should be reoriented to anterior commissure and then coregistered to its MRI (and, thus, coregistered to MNI-152) also. Notably, only reorientation requires manual manipulation. Then, the coregistered individual MRI was warped into MNI space using unified segmentation in SPM12. The transformation matrix in this warping were applied to the coregistered individual PET for anatomical standardization into MNI space (Klunk et al. 2015; Matsuda et al. 2021). Other software like FreeSurfer (https://surfer.nmr.mgh.harvard.edu/) and PMOD PNEURO (https://www.pmod.com/) are also practicable for the above spatial normalization. Meanwhile, individual MRI images are sometimes difficult to obtain due to contraindications, therefore, software like CapAIBL (https://milxcloud.csiro.au/) is invented for PET quantitation without MRI.

A number of factors would influence the measured SUVR, including but not limited to the used radiopharmaceuticals, acquisition time, target and reference regions, partial-volume correction, and reconstruction algorithms (Klunk et al. 2015). These factors impede the pooling of data across sites and comparison of studies. To address this issue, an international party has proposed the “Centiloid” scaling method to generate a standardized quantitative amyloid imaging measurement value (see later). It is worth noting that although there are many methods available, not all of them can be easily implemented in practice in most clinical settings due to limited technical resources, inability to obtain normal controls, etc. It is necessary to develop user-friendly automatic semi-quantitative tools with normal references to be applicable to more clinical scenarios.

In this section, we summarize important factors and directions in the quantification of amyloid PET imaging.

#### Reference Regions

In addition to the cerebellar GM, the classic reference region selected for amyloid PET semi-quantification include whole cerebellum, pons, subcortical white matter (SWM). Besides, a ‘composite reference region’ of the cerebellum with WM has also been prescribed in longitudinal analysis of florebetapir PET, which allows for more accurate detection of changes over time (Landau et al. 2015). Instead of the traditional fixed atlas-based reference regions as above, other methods based on data-driven approaches were also tested (Chen et al. 2015; Wang et al. 2021). The advantages and disadvantages of each reference are summarized in Table 3 (Bullich et al. 2017; Chen et al. 2015; Cho et al. 2020; Devous et al. 2018; Klunk et al. 2015; Wang et al. 2021). It is noted that amyloid PET images should be interpreted with care when the reference regions have the potential to be affected by specific factors. For example, cerebellum is usually involved in amyloid deposition in late-stage AD patients as well as those with specific genetic mutations (e.g., presenilin-1), in which cases it is recommended to confirm the presence of amyloid accumulation in cerebellum by visual inspection, or evaluate the Centiloid scale using different reference regions (see later).

**Table 3:** Characteristics of different reference regions in amyloid PET quantification

Optional reference region	Advantages	Disadvantages
CGM	Free of Aβ Same non-displaceable activity as the target area	The susceptibility to noise such as the nonspecific signal from the cerebellar peduncles and specific binding from adjacent cortical tissues Low sensitivity for signal due to its location at the edge of the scanner’s field of view Artifacts via truncation or attenuation correction of images due to the low position Amyloid deposition in cerebellum in patients with gene mutations and at advance stages
WC	Same as CGM Higher signal intensity and less susceptibility to noise compared to CGM Include tissue less vulnerable to edge and truncation effects compared to CGM	Almost the same as CGM
Pons	Same as CGM	Small size Sensitivity to head motion The poor performance of many normalization routines The susceptibility to scatter and truncation effects due to its location at the outer extremes of the field of view of an axial PET scanner
WC + B	Same as WC	Same as WC and Pons
SWM	Large region Locate approximately the same as target VOI in the axial field of view to reduce variability Represent the average uptake value of signal intensity, potentially leading to less noise More resistant to small degrees of misregistration during image quantification	Atrophy and vascular lesions White matter could play a specific role in amyloid compound uptake Affect by the combined effects of fibrillar Aβ and partial-volume averaging
PERSI-WM	The same as SWM PVE correction Individual based non-specific binding voxels with a lower intensity than other contaminated voxels	Same as SWM Individual MRI is required

*CGM* cerebellar gray matter, *WC* whole cerebellum, *WC* + *B* whole cerebellum plus brainstem, *SWM* subcortical white matter, *PERSI-WM* parametric estimation of reference signal intensity-white matter

#### Target VOI

While amyloid deposition usually spreads all over the brain, lateral temporal and frontal lobes, the posterior cingulate cortex/precuneus, and the parietal lobes form the vulnerable regions of amyloid deposition in typical AD. Therefore, a ‘composite-VOI’ including all vulnerable and particularly vulnerable areas is usually suggested as the target VOI. In addition, for ^18^F-flutemetamol PET scans, the VOI should also include the striatum. Templates of VOIs in standard space like Automated anatomical labeling atlas 3 (Rolls et al. 2020) and Maximum probability atlas (Hammers N30R83) (Hammers et al. 2003) could be used for VOI definition.

#### The Cut-off Value for Positivity

A cut-off value to determine amyloid positivity or negativity in clinical settings is desired to evaluate amyloid status more objectively. Though some studies reported the optimal cut-off values (Bullich et al. 2017; Landau et al. 2014; Landau and Jagust, 2015), applying such a threshold should be careful since the differences among compounds used and the calculation methods of SUVR invariably complicate the situation. Therefore, if one wants to refer to such a cut-off value, one should adhere strictly to the quantification method used to obtain the cut-off value. For instance, Alzheimer’s Disease Neuroimaging Initiative (ADNI) recommends the florbetapir cut-off as 1.11 when the SUVR is normalized by the whole cerebellum reference region, and the summary SUVR is a conventional (nonweighted) average across the four main cortical regions (frontal, anterior/posterior cingulate, lateral parietal, lateral temporal defined by Freesurfer) (Landau and Jagust 2015). For flutemetamol, when using the whole cerebellum as the reference region and using the global cortical target (CTX)-VOI consisting of frontal, temporal, parietal cortices, precuneus, anterior striatum and insular cortex for SUVR quantification, the recommended positive cut-off value is 1.13 (Matsuda et al. 2021). The cut-off for the florbetaben scan is 1.43 when the reference region is cerebellar GM and the SUVR is the mean of the six cortical regions (frontal, occipital, parietal, lateral temporal, anterior and posterior cingulate cortex regions) (Bullich et al. 2017). And the NAV4694 cut-off of amyloid positivity is 1.55 SUVR when the PET image is intensity normalized by cerebellar GM and corrected for partial-volume effect. The global NAV4694 SUVR is averaged with SUVRs in the precuneus, prefrontal, orbitofrontal, parietal, temporal, anterior, and posterior cingulate cortices (Therriault et al. 2021).

#### Centiloids Scale

Developed from SUVR, the “Centiloid” aims to offer comparable results across analysis techniques and tracers by linearly scaling the outcome data of any amyloid PET method to an average value of zero in “high-certainty” amyloid-negative subjects and to an average value of 100 in “typical” AD patients (Klunk et al. 2015). Standard processing and quantification workflows reconcile amyloid deposition regardless of the amyloid PET tracer used and the reference region chosen, thus making it more practical in clinical routine and multicenter studies. The “standard” method is suitable for ^11^C-PiB PET data acquired between 50 and 70 min after injection, and the “nonstandard” method could be used for ^11^C-PiB PET data with different scanning protocols as well as other amyloid tracers. A standard cortical VOI (CTX) that covers the areas of significant ^11^C-PiB tracer binding in AD and a whole cerebellum VOI to use as the reference region is available from the Global Alzheimer’s Association Interactive Network (GAAIN) website (http://www.gaain.org). So far, Centiloid scale is available for all amyloid tracers (^11^C-PiB (Klunk et al. 2015), florbetapir (Navitsky et al. 2018), flutemetamol (Battle et al. 2018), florbetaben (Rowe et al. 2017), and NAV4694 (Rowe et al. 2016)).

#### Establishment of Databases

Although cut-off SUVR value for amyloid positivity are available for different ligands from publications (as mentioned above), the differences in imaging acquisitions and reconstructions among centers would to some extent make the common cut-off value not exactly suitable for each center (Bourgeat et al. 2021), let alone the inconsistency of the SUVR analysis methods. Meanwhile, though without clear evidence, the racial differences should not be ignored. Therefore, for clinical rounite and multicenter study, Centiloid scale is recommended for appropriate transformations (Klunk et al. 2015). At the same time, while Centiloid scale is based on fixed VOIs and reference region to offer a global assessment of amyloid deposition, the analyses with specific VOIs and different reference regions are important, especially in longitudinal follow-up. If feasible, it is recommended that each center, or each region/country to establish its own dataset for healthy control subjects and AD patients within different stages, which would help to validate of the Centiloid approach and other standard pipeline established in the literature. Moreover, if possible, it is recommended to make the final interpretation based on comparison with multiple preselected sets of healthy control subjects who are matched in genetic status with the patient and scanned exactly in the same way, since cerebellum is usually involved in amyloid deposition in autosomal dominant AD (i.e., presenilin-1 mutations) (Giau et al. 2018; Lu et al. 2021).

#### Voxel-Wise Analysis

Besides the VOI-based method, voxel-wise analysis is also widely used in PET brain images. It could compare the difference between two groups, or one subject and one control group directly in the whole brain after normalization. As voxel-based, it could detect the slight abnormality without the restriction of pre-defined VOI (Akamatsu et al. 2019; Planton et al. 2020).

#### Partial Volume Effect (PVE) Correction

PVE is one of the biological factors may contribute to within-subject amyloid PET signal variability. This phenomenon refers to that because of the unavoidable progressive cortical atrophy in AD, the loss of tissue volume can result in a drop in the amyloid signal in the absence of change in amyloid burden. Therefore, when quantifying, especially in longitudinal studies and clinical treatment trials with amyloid PET changes as endpoints, partial-volume correction methods which attempt to recover the true value of the PET signal by estimating and adjusting for spillover contributions from neighboring white, CSF, or other gray matter tissue needs to be considered (Quarantelli et al. 2004; Schmidt et al. 2015). Several studies have reported that applying PVE correction could improve the quantitative analysis of amyloid PET (Brendel et al. 2015; Rullmann et al. 2016, 2020; Teipel et al. 2021). It is worth noting that the PVE correction is highly method-dependent, and its use must always be applied with caution, including recognizing that the threshold for positivity will vary with method to be applied, and that when using MRI-based PVE correction, the reference MRI must be acquired on the same scanner to be reliable. So far, no consensus on when and which correction to use is available.

#### AI-Based Analyses

Artificial intelligence (AI) has been a hot topic of the recent emerging trends in imaging research. Though far away from a routine clinical application, the preliminary findings show great potential for a better understanding PET images. For instance, with AI methods, the definition of amyloid positivity established an excellent agreement with the visual assessment (Kim et al. 2021; Thurfjell et al. 2014; Vandenberghe et al. 2013); the quantification of amyloid burden was improved by removing non-specific bindings (Liu et al. 2021); PET data could be harmonized by generating imputed amyloid PET images of one radiopharmaceutical from the images of another (Shah et al. 2022). AI also achieved the amyloid PET staging (Kim et al. 2020). The machine learning methods are potentially helpful in developing algorithms to get sufficient information from those ultra-low-count (to reduce the scan time) and ultra-low-dose (to reduce the injected radiotracer dose) amyloid PET, which could help to decrease image quality degradation caused by patient’s head motion without sacrificing image quality and diagnostic power, enhancing the patient-throughput of imaging room (Chen et al. 2019, 2020, 2021).

## Data Security

To guarantee the integrity and security of imaging/clinical databases and maintain the confidentiality of protected health information of subjects, all related material should be kept in a dedicated place by a dedicated group. All data used for teaching and research purposes must first obtain the informed consent of the examinee and anonymization is compulsive. Data transmission and sharing need to be subject to relevant regulations.

## Conclusion

With the increasing availability of amyloid PET and the continuous development of clinical trials for drug development targeting amyloid, together with the complexity of amyloid PET itself, detailed and standardized imaging procedures for the patient assessment process are critical in clinical practice. Only qualified physicians and technologists should be allowed to carry out corresponding work. Clinical assessment for patient screening should adhere to the indications and non-indications of amyloid PET. An establishment of a standardized radiopharmaceutical synthesis and quality control process and a standardized image acquisition and processing methods provide excellent quality amyloid PET study. The final report should integrate all the information of patient, not only by the amyloid PET but also by the other imaging modalities and clinical data. Besides, CSF and plasma biomarkers are available for amyloid detection now (Janelidze et al. 2021; Nakamura et al. 2018); the former is invasive with low accessibility, and the latter is cheap and noninvasive. While plasma amyloid-β biomarkers could facilitate broader clinical access and efficient population screening, amyloid PET with detailed distribution information showing the disease heterogeneity (Toledo et al. 2019; Wolk et al. 2012) could further aid in differentiation (i.e., amyloid-positive subcortical vascular cognitive impairment) (Jang et al. 2018) and progression prediction (Yoon et al. 2021), as well as provide additional information such as estimation of the likelihood of tau positivity in amyloid-positive individuals (Raman et al. 2022). Given the usefulness of high-availability ^18^F-FDG PET in differential diagnosis of dementia, we agree with the diagnostic algorithm by an interdisciplinary group of experts comprised of nuclear medicine physicians, radiologists, neurologists, geriatricians, psychiatrists, clinical and basic neuroscientists, and patient advocates in 2020 (Chételat et al. 2020) that amyloid PET is recommended to be in the second place in the following clinical settings: (1) elderly patients (older than 80 years) due to the not low amyloid positivity rate in the elderly with normal cognition (Jansen et al. 2015); (2) patients for whom AD is not the single most probable suspected diagnosis because amyloid is less informative for differential diagnosis of dementia. Therefore, physicians should select the appropriate testing protocol according to the actual situation.

## Data Availability

Not applicable.
